# Medical students’ perceptions of the patient-centredness of the learning environment

**DOI:** 10.1007/s40037-016-0317-x

**Published:** 2016-12-16

**Authors:** Mark V. Wilcox, Megan S. Orlando, Cynthia S. Rand, Janet Record, Colleen Christmas, Roy C. Ziegelstein, Laura A. Hanyok

**Affiliations:** 1 Department of Obstetrics, Gynecology, and Reproductive Sciences, University of California, San Francisco, CA USA; 2 School of Medicine, Johns Hopkins University, Baltimore, MD USA

**Keywords:** Patient-centredness, Learning environment, Empathy, Medical student

## Abstract

**Background:**

Patient-centred care is an important aspect of quality health care. The learning environment may impact medical students’ adoption of patient-centred behaviours.

**Methods:**

All medical students at a single institution received an anonymous, modified version of the Communication, Curriculum, and Culture instrument that measures patient-centredness in the training environment along three domains: role modelling, students’ experience, and support for patient-centred behaviours. We compared domain scores and individual items by class year and gender, and qualitatively analyzed responses to two additional items that asked students to describe experiences that demonstrated varying degrees of patient-centredness.

**Results:**

Year 1 and 2 students reported greater patient-centredness than year 3 and 4 students in each domain: role modelling (*p* = 0.03), students’ experience (*p* = <0.001), and support for patient-centred behaviours (*p* < 0.001). Female students reported less support for patient-centred behaviours compared with male students (*p* = 0.03). Qualitative analysis revealed that explicit patient-centred curricula and positive role modelling fostered patient-centredness. Themes relating to low degrees of patient-centredness included negative role modelling and students being discouraged from being patient-centred.

**Conclusions:**

Students’ perceptions of the patient-centredness of the learning environment decreased as students progressed through medical school, despite increasing exposure to patients. Qualitative analysis found that explicit patient-centred curricula cultivated patient-centred attitudes. Role modelling impacted student perceptions of patient-centredness within the learning environment.

## What this paper adds

Patient centredness is an essential component of high quality health care and should be present within the medical school learning environment. This paper aims to understand and qualitatively characterize the patient-centredness of the medical school learning environment and how it may change based on curricular year. Students reported the environment’s patient-centredness decreased as they progressed through their education. Female students reported less support for patient-centred behaviours. Students commonly observed positive and negative role modelling impacting the learning environment. This suggests that patient-centredness should be explicitly taught and that focus should be on the attitudes and behaviours modelled by clinicians in the clinical years.

## Introduction

Patient-centred care is a model for collaborative medical interactions. Components of patient-centred care include: 1) exploring both the disease and the patient’s illness experience, 2) understanding the whole patient, 3) finding common ground between patient and physician about priorities and management, 4) incorporating prevention and health promotion, 5) enhancing the patient-physician relationship, and 6) being realistic about time and resources [1]. The patient-centred model is associated with improved patient outcomes [2], a finding that prompted the Institute of Medicine to highlight patient-centred care as one of six core aspects of high quality health care [3]. Medical schools have developed curricula to teach patient-centred practices. However, informal aspects of the learning environment may impact medical students’ adoption of patient-centred beliefs and behaviours. These include the interpersonal, unscripted interactions that constitute the informal curriculum, and the cultural structures that define the hidden curriculum [4].

The Communication, Curriculum, and Culture instrument is a validated tool that quantitatively measures patient centredness in medical school learning environments. This 29-item questionnaire produces three subscale scores that quantify the extent to which attending physicians and house staff model patient-centred behaviour, the relative patient-centredness of students’ experiences, and perceived support for students’ patient-centred behaviours [5]. The instrument was developed using a process of item writing and refinement, item selection, and determination of reliability and validity as described by the authors [5]. Although the instrument was found to be reliable and valid, a notable limitation is that there is no gold standard for the definition of patient centredness, and for that reason expert opinion was used for item writing and refinement. Our objective was to use a modified version of the Communication, Curriculum, and Culture instrument that pertained to the experiences of both preclinical and clinical students to evaluate the patient centredness of aspects of medical school training. We sought to measure the perception of the patient-centred nature of the learning environment, determine whether that perception varied by year of training or gender, and elucidate the types of experiences students perceived to have high or low degrees of patient-centred.

## Methods

### Study methods and measure

An online anonymous survey was sent to all 498 medical students at the Johns Hopkins University School of Medicine in Baltimore, Maryland. The survey, sent in the late spring and therefore near the end of the class year, included the Communication, Curriculum, and Culture instrument [5], a validated instrument that quantitatively measures the patient-centredness of the medical study learning environment. This instrument measures three domains on 5‑ or 7‑point Likert scales. Role modelling was scored by averaging of ten items measured on a 7-point scale indicating frequency of patient-centred observations (i. e. ‘Please indicate how often you observed chief residents communicate concerns, and interested in patients as unique persons.’) The students’ experience domain was measured by the mean of 11 items, with 4 items composing the ‘learning relationships’ dimension being reversed scored, on a 5-point scale indicating how often he/she has experienced a similar situation (i. e. ‘You hear students telling stories about patients. These stories tend to portray patients as diagnoses rather than unique human beings.’) Support for students’ own patient-centred behaviours was measured using a 5-point Likert scale from ‘completely encouraged’ to ‘discouraged.’ ‘Respondents’ are asked to rate the response that was received from instructors by filling in the blank (i. e. ‘In general, when I made an effort to develop rapport with patients, my instructors ... me.’) A higher score represented greater patient-centredness in the role modelling and the support for students’ behaviours domains; a lower score represented greater patient-centredness in the students’ experiences domain.

Because the wording of the original Communication, Curriculum, and Culture instrument pertains only to medical students during their clinical years (i. e., years 3 and 4), we created an additional version of the instrument for preclinical students (years 1 and 2) in which titles referenced in particular items (e. g. *chief residents, senior residents, or interns*) were made relevant (e. g. *course faculty or clinical mentors*). We also added two qualitative survey items to characterize students’ personal patient-centred experiences through the following questions: 1) Describe an encounter or experience that reflected a high degree of patient-centredness, and 2) Describe an encounter or experience that reflected a low degree of patient-centredness. We gathered information on respondents’ gender (female, male) and class year [1–4].

This work was approved by the Johns Hopkins University Institutional Review Board (Study number CR00009001) and was carried out in accordance with the Declaration of Helsinki.

### Study setting

The curriculum of the Johns Hopkins University School of Medicine is called the Genes to Society curriculum (available online at http://www.hopkinsmedicine.org/som/curriculum/genes_to_society/curriculum/interactive_map.html) and encompasses students’ full four years of medical school. This curriculum emphasizes an integrative approach to patient care. For this reason, clinical experiences begin early, and include a longitudinal clerkship in years 1 and 2. However, the frequency of clinical experiences increases significantly in years 3 and 4, and the bulk of clinical rotations occur during the second two, traditionally named ‘clinical’ medical school years. Clinical rotations occur in the ‘real world’ clinical environment as opposed to classroom and simulation experiences that are planned and can be specifically created to emphasize patient-centred skills.

### Study analysis

The modified Communication, Curriculum, and Culture instrument quantitatively measures three patient-centred domains on 5‑ or 7‑point Likert scales: role modelling, students’ experience, and support for students’ own patient-centred behaviours. The distributions of scores in each domain were within acceptable limits of skewness. Mean differences in domain scores were compared by gender and class year (years 1 and 2 and years 3 and 4) using independent groups t‑tests (Microsoft Excel version 14.5.3). In addition, we were interested in determining how often students responded in a desired patient-centred fashion by class year. For the role modelling domain with a 7-point Likert scale, a response of 6 or 7 was considered desired, and for the students’ experiences and support for student behaviour domains with a 5-point Likert scale, a response of 4 or 5 was considered desired. For each item we computed chi square tests to examine whether there were differences in the proportion of patient-centred responses between year 1 and 2 vs. 3 and 4 students. The confidence level of all analyses was 0.05.

Qualitative responses were evaluated using an editing style analysis [6]. Three study team members (MW, MO, and LAH) independently reviewed students’ survey responses to identify thematic categories and subcategories prior to discussing this with the other two members. Year 1 and 2 and year 3 and 4 responses were reviewed together. Individual coding strategies were developed by each team member to identify themes. Themes were compared and those common to all team members were accepted. Two investigators were medical students at the time of the study; one investigator was a faculty member. All three investigators participated in each step of the analysis. All decisions were made by team consensus.

## Results

Thirty-one percent of students (156/498) completed the survey; 49% of respondents were female and 51% were male. The overall student body composition is approximately 50% female. The response rates for year 1 and 2 (preclinical) and year 3 and 4 (clinical) students were similar, with 77 preclinical and 79 clinical student responses.

Preclinical students reported significantly greater patient centredness than clinical students in all three domains. For role modelling (preclinical students’ mean = 5.27 vs. clinical students’ mean = 4.96, the 0.32) difference in means between groups indicated significantly higher ratings among preclinical students (95% CI [0.03, 0.61], *p* = 0.03). On students’ experience ratings, preclinical students ratings (mean 2.48) were significantly lower than clinical student ratings (mean = 2.79, 95% CI [−0.45, −0.15], *p* < 0.001). In the domain measuring support for students’ own patient-centred behaviours, preclinical student scores (mean 4.24) were significantly higher than clinical student scores (mean = 3.76, 95% CI [0.20, 0.77], *p* = 0.001). Aggregated across all four years, female students (mean 4.15) reported lower support for their own patient-centred behaviours compared with male students (mean = 3.83, 95% CI [0.04, 0.62], *p* = 0.03). There were no significant differences in mean scores by gender in role modelling or student experiences domains year 1/2 and 3/4 student responses were also compared by item. Multiple significant differences were observed (Table 1). Some student experiences differed based on year in school. Year 3 and 4 students reported patients were perceived or treated as objects more often than year 1 and 2 students. Clinical students also felt they received less feedback about their bedside manner and listening skills than did year 1 and 2 students. This correlates with the domain of support for patient-centred behaviours as year 3 and 4 students felt less supported than year 1 and 2 students when acting in a patient-centred manner. Notably, year 1 and 2 students reported greater patient-centredness for every item in which a significant difference was found.

**Table 1:** Chi square comparisons of preclinical (year 1/2) and clinical (year 3/4) students’ responses to each item on the Communication, Curriculum and Culture instrument

**Category**	**Domain**	**Item summary**	**Percent respondents answering in desired patient-centred fashion**		***p*** **-value**
			**Year 1/2 in %**	**Year 3/4 in %**	
**Role modelling**	Course faculty	Communicate concern and interest in patients as unique persons	49	43	0.429
		Encourage patients’ participation in their own care	39	33	0.431
		Take seriously patients’ concerns about their conditions or care	56	49	0.418
		Develop good rapport with patients	58	44	0.077
		* Explore emotional aspects of patients’ illnesses	34	16	**0.013**
	Clinical mentors	* Communicate concern and interest in patients as unique persons	57	37	**0.011**
		Encourage patients’ participation in their own care	44	33	0.149
		Take seriously patients’ concerns about their conditions or care	53	44	0.264
		* Develop good rapport with patients	65	48	**0.034**
		* Explore emotional aspects of patients’ illnesses	39	22	**0.018**
**Students’ experiences**	Patients as objects	* Preceptor overheard referring to patient as a diagnosis	5	46	**<0.001**
		* Little attention paid to social history	12	37	**<0.001**
		Patient waits longer than necessary	3	11	0.056
		* Students overheard portraying patients as diagnoses rather than unique human beings	16	39	**0.001**
		* Patient’s case discussed in front of patient as if the patient weren’t there	4	24	**<0.001**
	Learning relationships	Students’ stories portray how a patient encounter affected the student(s) personally	58	59	0.894
		* Student provided with feedback on bedside manner	39	86	**<0.001**
		* Student provided with feedback on listening skills	29	67	**<0.001**
		Advice given to students emphasizes the importance of good communication skills with patients	70	75	0.525
	Bad news	Student asked to deliver bad news without any teaching or discussion about a caring approach	94	96	0.445
		Student asked to answer patient’s questions about bad news without any teaching	92	91	0.809
**Support**	–	* Student felt encouraged when an effort was made to develop rapport with patients	88	73	**0.018**
		* Student felt encouraged when an effort was made to know patients as unique persons	83	66	**0.013**
		* Student felt encouraged when an effort was made to legitimize patients’ concerns	88	67	**0.001**

Despite their disparate characterization of the patient-centredness of their learning environment, students from all class years qualitatively described experiences which pin our analysis had similar themes emerge regarding experiences with high (Table 2) and low (Table 3) degrees of patient centredness. When asked about highly patient-centred experiences, the following themes emerged from students’ responses: (1) explicit patient-centred teaching in the curriculum, (2) positive role modelling, and (3) independent time to interact with patients all foster patient-centredness. Themes that were identified from students’ descriptions of experiences with low degrees of patient-centredness are as follows: (1) negative role modelling (with sub-themes of ignoring patient concerns, poor communication, and lapses in professionalism), (2) students discouraged from performing patient-centred behaviours, and (3) objectification/lack of humanism.

**Table 2:** Themes with representative quotes from medical student experiences that were highly patient-centred

	**Year 1/2 Medical students**	**Year 3/4 Medical students**
Explicit teaching of patient-centred care	*Patient-centred skills/strategies are taught and assessed in standardized or guided clinical contexts* ‘In CFM (Clinical Foundations of Medicine), teaching open-ended questions was a huge eye-opener for me coming into medical school.’ ‘Most of CFM, where we interviewed and got to know patients without any real medical knowledge. Placing the course early in our training emphasized patient-centeredness (because that’s all we could do).’ ‘CFM final interview-testing preceptor was very positive about my interviewing style which diverged from “traditional” interviewing and was much more conversational. He liked how it drew a lot of information from the patient and helped to develop rapport, even though I missed a decent chunk of the history.’	*Teams created to be or rounds intentionally structured to be patient centred* ‘On Medicine, chief rounds at Bayview. Group really gets to know the patient as a person first, before starting to talk about their illness.’ ‘I helped take care of a gentleman on the Aliki (Green) team during my medicine rotation. He was admitted for pre-renal AKI secondary to volume depletion in his nursing home. During attending rounds, I was encouraged to present the entire admission history and physical to the patient and his wife as patient-centred rounds. Though the patient had altered mental status, the patient’s wife was extremely grateful for having been included as part of the care team. It was one of the most memorable and rewarding experiences of medical school thus far.’
Role modelling or implicit teaching of patient-centred care	*Students are influenced by practitioners who model patient-centred approaches and interactions* ‘I have so many stories of amazing patients with complex lives, situations, challenges, and achievements. My longitudinal clerkship (LC) preceptor was really good at showing how to be friends with your patient while still maintaining the professionalism of the relationship.’ ‘CFM was amazing. My advisor very much values patient-centred care and consistently modelled it for us.’	*Students are influenced by practitioners who model PC approaches and interactions* ‘I worked with my attending who was just incredibly understanding and compassionate with parents of a newborn who were unwilling to vaccinate their child … My attending was truly professional, non-judgmental, and compassionate throughout the encounter.’ ‘ED resident who would squat next to the bedside of every patient so he would be on eye level.’
Other factors	*Students need opportunities to independently practice implementing patient-centred skills* ‘During LC when taking 30 min interviews independent from supervision. Those were the times when there was the least pressure to get clinical information and then stop, but rather to get to know the patient as a person.’ ‘LC was by far the most patient-centred experience I’ve had, simply because of the longitudinal opportunity to work one-on-one with patients and develop my own communication skills with guidance and support from my preceptor.’	*Significant time to talk with patients* ‘Having the time to talk to patients one on one about their disease and how it has affected their abilities to go on the rest of their lives.’ *Interprofessionalism* *‘*Working with patients and their families, consistent interprofessional collaboration to help patients with issues outside the scope, strictly defined, of their ‘medical’ issues.’

**Table 3:** Themes with representative quotes from medical student experiences that were least patient-centred

	**Year 1/2 Medical students**	**Year 3/4 Medical students**
Negative role modelling	*Ignore patient concerns* ‘Although he never asks the patients about anything besides symptoms … one patient brought up out of the blue that she was stressed about the fact that her husband was in the end stages of lung cancer. My preceptor’s response was to continue asking about side effects from an antibiotic.’ *Failure to include the patient in team discussion or teaching* ‘My longitudinal clerkship (LC) preceptor frequently talked to me about the patient while the patient was in the room, as if the patient were not in the room.’ *Lapses in professionalism* *‘*When I am in the hospital (shadowing, on a rotation, etc.), I often overhear nurses, residents, and/or attendings make distasteful comments about patients or families who are difficult to work with. Sometimes … within earshot of the patients and families.’	*Ignore patient or other providers’ concerns* *‘*Medical professionals ignoring patients’ own words or nodding and seeming to agree when the patient is speaking, but then proposing a different plan of action without acknowledging the patient’s wishes for something different’ ‘Attending kind of side-stepped patients concerns and passed the buck off to her medical oncologist rather than take the time to explain things.’ *Lapses in professionalism* *‘*13 year old girl came in to paediatric ED with increased vaginal bleeding, was uncomfortable seeing me (as a male) and requested a female provider … 2 female PAs were available; the attending, laughed, said she would have to be punished and wait for 20 mins for refusing to talk with me and said to the patient should get over it, she was just having a period. PAs and attending then continued to online shop for 20 mins before one of the PAs went to see the patient.’
Students discouraged from being patient-centred	*Regarding the social history* ‘The physicians chosen for Preceptor Skills repeatedly told me that Social History is a waste of time and docked my write-up every time I had that section.’ *Discounting idealism* ‘Basically every lecture where physicians tell us about how one day we will get out of the false pretences of medical school and into the ‘real world’ of medicine.’	*Spend as little time as possible with patients* ‘Multiple instances on surgery when I was encouraged to spend less than 3 min with the patients if at all possible.’
Other factors	*Objectification* ‘Anatomy was horrible. The instructors saw the cadavers as pieces of objects instead of real, dead people. I was disturbed.’ *In time-sensitive situations, attention to a patient’s background, story, or social history is limited* ‘Some of my LC experiences have been severely time limited such that I have been forced to ask only “medically relevant” questions that completely forgo engaging the patient as more than a diagnosis.’	*Objectification* ‘Patients were discussed by disease with many negative comments about their BMI.’ ‘Patients became fractures and operations; there was very little interaction with patients (the most interaction was waking the patient up at 4:45 AM to make sure he/she could wiggle his/her toes).’

## Discussion

In this mixed methods approach to understanding the learning environment at one institution, students’ reports of patient-centredness decreased as they progressed through medical school and, curiously, as exposure to patients increased. As has been noted previously in the literature [7], there has been relatively little research on factors that promote or inhibit patient-centredness among medical students. This underscores the importance of soliciting students’ opinions and ideas based on their experiences in medical school. Our qualitative analysis of students’ responses indicated that formal learning methods in courses and on clinical teams contributed to patient-centred learning experiences. This corresponds with previous students who suggest that patient-centred care can be taught in medical curricula [8–10], but does not exclude the importance of other factors.

Our study affirms that both positive and negative role modelling strongly affect students’ perception of the learning environment. Some of the most detailed qualitative survey responses involved students’ recollections of particular exchanges between clinical mentors and patients. This supports a number of prior evaluations of the undergraduate medical education learning environment and has been described as a major component of the ‘hidden curriculum’ [4, 11–13]. Although the learning environment significantly differs among US medical schools [14], it has been shown repeatedly over the last 50 years that medical students lose empathy and humanism and become more cynical and less patient-centred the further they go in their training [15–19]. Our results suggest that role modelling by clinical mentors plays a major role in this trend.

Female students in our study reported less support than male students for their own patient-centred behaviours during medical school. Previous research demonstrates that female students consistently display greater empathy and patient-centredness than male students during patient encounters [18, 20]. It is possible that female students do not feel as supported in patient-centred behaviours as male students do because instructors do not see as much room for improvement in their patient-centred skills. Alternatively, this result may reflect an implicit recognition of the above noted gap between female and male students as female students feel social or professional pressures to decrease their patient-centred behaviours. Additionally, we do not know the gender of the faculty and resident trainees with whom students worked. It is possible that the gender of those supervising physicians may have affected these findings.

The major limitation to our study is the small sample size. Approximately one-third of medical students responded to the survey and it is possible that these students had uniquely strong views on the topic of patient-centredness so that their experiences may not accurately represent the larger student body. Additionally, our study population is limited to one institution during one academic year and our results may not correspond with the entirety of undergraduate medical education. Furthermore, it is difficult to know what results are clinically or educationally significant using the Communication, Curriculum, and Culture instrument. It is possible that although we have found statistically significant differences in learners’ experiences, this does not have a clinically or educationally significant impact on them. Fewer students responded to qualitative survey items than quantitative questions and it is possible that our results give more of a voice to students with outlying experiences.

## Conclusion

In conclusion, we found that student assessments of the patient-centredness of the learning environment decreases as students’ progress from years 1/2 to years 3/4 of medical school and, curiously, as exposure to patients increases. Students describe positive influences on the learning environment, which include explicit curricula to teach patient-centred care, and role modelling of patient-centred behaviours. They noted that negative role modelling and discouragement from supervisors were negative influences on the patient-centredness of the learning environment. Since patient-centred care is an important attribute for physicians and is a core aspect of high quality health care [3], these findings suggest a need to explicitly teach patient-centredness throughout the medical school curriculum and to focus specifically on the attitudes and behaviours modelled by more senior clinicians in the clinical years. Additionally, at our institution, we found that female medical students reported decreased support for patient-centred behaviours as compared with male medical students. This observation should be further explored to better understand the underlying reasons for this report. Standardized evaluations of institutional learning environments are necessary to effectively plan and execute interventions aimed at increasing the patient-centred behaviours of current and future clinicians.
